# Optimising diagnosis and post-diagnostic support for people living with dementia: geriatricians’ views

**DOI:** 10.1186/s12877-022-02814-0

**Published:** 2022-02-19

**Authors:** Elise Mansfield, Jamie Bryant, Balakrishnan R. Nair, Alison Zucca, Ranjeev Chrysanth Pulle, Rob Sanson-Fisher

**Affiliations:** 1 Health Behaviour Research Collaborative and Priority Research Centre for Health Behaviour, School of Medicine and Public Health, College of Health, Medicine and Wellbeing, University of Newcastle, Callaghan, NSW Australia; 2 Hunter Medical Research Institute, New Lambton Heights, NSW Australia; 3 John Hunter Hospital, Hunter New England Local Health District, New Lambton Heights, NSW 2305 Australia; 4 School of Medicine and Public Health (Medical Education and Professional Development), University of Newcastle, Callaghan, NSW 2308 Australia; 5 Internal Medical Services, The Prince Charles Hospital, Chermside, QLD 4032 Australia

**Keywords:** Dementia, Alzheimer’s disease, Geriatricians, Health care providers, Diagnosis, Quality of care, Guidelines

## Abstract

**Background:**

Providing a timely and accurate diagnosis of dementia and delivering appropriate support following a diagnosis are essential to allow individuals and their families to plan for the future. Recent studies suggest that provision of diagnosis and post-diagnosis support is suboptimal. This study explored geriatricians’ views about strategies to improve quality of care across these domains.

**Methods:**

An anonymous online survey of geriatricians and advanced trainees in one Australian state was conducted. An Expert Advisory Group of geriatricians, behavioural scientists and consumers proposed strategies to improve quality of care in relation to diagnosis and post-diagnosis support for people with dementia, which formed the survey items. Potential strategies were guided by, but not limited to, dementia and chronic care guidelines. Participants were asked the extent to which they agreed that implementing each of the proposed strategies would improve the quality of dementia care.

**Results:**

Of 59 participants (response rate 42%), all agreed that improving accessibility of geriatricians would improve the accuracy and timeliness of diagnosis. Over 90% were supportive of strategies to improve capacity of general practitioners to accurately diagnose dementia. Between 97-100% agreed that information provided following diagnosis should encompass symptom progression, treatments, psychological supports, and advance care planning. Just over two-thirds thought that life expectancy should be discussed at this time. There were high levels of support for strategies already included in existing dementia care guidelines, however geriatricians also agreed with a range of possible strategies not currently included in guidelines.

**Conclusions:**

Geriatricians perceive that timeliness and accuracy of dementia diagnosis may be improved by increasing access to geriatricians and training general practitioners in diagnosing dementia. They also believe it is appropriate to provide information at the time of diagnosis across a comprehensive range of areas, including potentially sensitive topics such as advance care planning. Future studies should explore the views of other groups of health care providers and consumers about these approaches. The strategies proposed should be considered for inclusion in future dementia care guidelines.

**Supplementary Information:**

The online version contains supplementary material available at 10.1186/s12877-022-02814-0.

## Background

Identifying symptoms of dementia and receiving a diagnosis as early as possible can provide those diagnosed and their families with more opportunities to learn about the condition and organise appropriate support [1]. However, in Australia there is an average of 3.1 years between first noticing dementia symptoms and receiving a confirmed diagnosis [2]. While consumers report wanting to know as soon as possible if they had dementia [3, 4], some research has shown that only half of patients who receive positive dementia screening results wish to receive further assessment [5]. This discrepancy highlights the importance of considering individual consumer preferences in obtaining a diagnosis. As opposed to early diagnosis, timely diagnosis refers to delivery of a diagnosis at the right time for an individual, taking into consideration their circumstances and preferences [6].

Increased expectations on providers to improve timeliness of a diagnosis of dementia may lead to less thorough symptom assessment and inaccuracies in diagnosis [1]. Given symptoms of dementia overlap with other conditions such as depression, there is potential for either missed dementia diagnoses (i.e. false negatives), or misdiagnosis of other conditions as dementia (i.e. false positives) [7]. Missed diagnoses and misdiagnosis can deprive the individual and their families of opportunities to obtain needed dementia-specific support and care, place unnecessary burden on the individual diagnosed and their families, and may lead to inappropriate management of the underlying condition [1]. Therefore the drive to increase timeliness of diagnosis should be accompanied by strategies to improve diagnostic accuracy. Several patient, provider and system level barriers exist to obtaining a timely and accurate diagnosis, including patients’ reluctance to acknowledge symptoms [8], lack of training and confidence among health care providers to accurately diagnose dementia [8], and lack of accessibility of specialist providers in less populated areas [9].

Timely and accurate diagnosis of dementia is only beneficial if the individual diagnosed and their family are equipped with the information and support they need to cope with the impacts of the diagnosis, make decisions about care and plan for the future [10]. This may include advice on obtaining practical assistance (e.g. housework, transport), social and emotional support (e.g. referrals to support groups) and planning for future decline in capacity (e.g. appointing a substitute decision maker). However, people with dementia and their support persons report a range of unmet needs for information at this time [11, 12]. In our recent study (unpublished), over half of carers were not offered referral to relevant support services such as counselling or community dementia support organisations at the time of diagnosis. Consequently, the drive for more timely and accurate diagnosis of dementia should include efforts to optimise post-diagnosis support.

Geriatricians are health care providers who specialise in the diagnosis and management of conditions that occur in older people, including dementia. In Australia, primary care providers are usually the first point of contact when symptoms of dementia arise. The primary care provider may take responsibility for assessment and diagnosis of dementia, or may refer on to a specialist such as a geriatrician, psychogeriatrician, neurologist or psychiatrist [2] for further investigation or confirmation of a suspected diagnosis. Most tertiary hospitals have multidisciplinary memory clinics that are available for the assessment of people with suspected dementia [9]. This study explores geriatricians’ views on the strategies needed to achieve optimal diagnosis and post-diagnosis support to people with dementia in Australia.

## Methods

### Design

This was a cross-sectional online survey conducted in one Australian state. The University of Newcastle Human Research Ethics Committee approved the study (H-2017-0283).

### Participants

Eligible geriatricians were members of the Australian and New Zealand Society for Geriatric Medicine (ANZSGM) in one Australian state, and were currently practising as a qualified geriatrician or advanced trainee. In Australia, an advanced trainee is an individual who has passed the basic physician examination in internal medicine and is undertaking a 3-year training program to become a geriatrician. The ANZSGM is the professional society for geriatricians and other practitioners in Australia and New Zealand with an interest in medical care of older people.

### Recruitment and data collection

All eligible individuals received a personalised invitation to participate from the President of the state division of the ANZSGM which included a copy of the participant information sheet and a link to the anonymous online survey. Completion of the survey was taken as voluntary consent to participate. Reminder emails were sent by the President four and six weeks following the initial invitation. Data were collected between June-December 2019 inclusive.

### Measures

A study-specific survey was developed (see Additional file 1). An Expert Advisory Group comprising geriatricians, behavioural scientists and consumers was asked to propose strategies to improve quality of care in relation to diagnosis and post-diagnosis support for people with dementia and their support persons. In developing strategies, the Expert Advisory Group were asked to consult Australian guidelines for dementia care [10], and guidelines for diagnosis and management of other chronic diseases (e.g. cancer). In proposing strategies, Expert Advisory Group members were asked to put forward suggestions that would have the highest likelihood of improving outcomes for people with dementia and their support persons. The proposed strategies were drafted into survey items. The draft survey was reviewed by the Expert Advisory Group for acceptability and utility for improving quality of dementia care.

The final survey included 19 items grouped into two domains: improving accuracy and timeliness of dementia diagnosis (7 items); and provision of information at the time of diagnosis to people with dementia and their support persons (12 items). The latter domain included items related to the type of information that should be delivered (e.g. “The benefits and risks of available treatment options”), as well as how the information should be delivered (e.g. “Information about dementia in multiple formats, including written and web-based, according to their preferences”). One of the items included in the accuracy and timeliness of diagnosis domain, and six of the items in the provision of information domain were based on recommendations included in Australian guidelines for dementia care (see Table 1). Items were presented as a series of statements, with participants indicating their level of agreement with each on a five-point Likert scale: Strongly Agree/Agree/Disagree/Strongly Disagree/Unsure. The terms ‘general practice’ and ‘general practitioner’ were used throughout the survey as these are the accepted terms for primary health care settings and primary health care providers, respectively, in the Australian context. Practice nurses are qualified nurses who work collaboratively with general practitioners in primary care settings. Demographic data collected included qualifications, number of years practicing, and number of people with dementia seen in the past month.

**Table 1:** Proportion of participants (*n*=59) that selected Agree or Disagree/Unsure for each item, ranked in descending order

	Based on Australian guidelines^a^	Agree *N* (%)^b^	Disagree/ Unsure *N* (%)^b^
**Improving accuracy and timeliness of diagnosis** To improve the accuracy and timeliness of dementia diagnosis there is a need for:			
Individuals to have access to a geriatrician in their local area	N	59 (100)	0 (0)
General practitioners to receive training in accurately diagnosing dementia	N	57 (97)	2 (3)
Increased community awareness about early symptoms of dementia and what to do if symptoms are noticed	N	56 (95)	3 (5)
75+ health assessments for general practice patients to include a standardised measure of cognitive impairment	N	54 (92)	5 (9)
Undergraduate medical students to receive training and rigorous skills assessment for dementia diagnosis	Y	52 (88)	7 (12)
Involvement of dementia-trained general practice nurses in the diagnosis of dementia	N	50 (85)	9 (15)
Consultations with geriatricians to be more affordable	N	47 (80)	12 (20)
**Provision of information at diagnosis** At the time of diagnosis, people with dementia and their support person/s should be provided with information about:			
The benefits and risks of available treatment options	Y	59 (100)	0 (0)
Lifestyle modifications that may improve health or quality of life	N	59 (100)	0 (0)
How to access psychological support or counselling	Y	59 (100)	0 (0)
Appointing a substitute decision maker(s) (e.g. Enduring Guardian and Enduring Power of Attorney)	N	59 (100)	0 (0)
Symptoms of dementia which may occur in future	Y	57 (97)	2 (3)
The benefits and process of making an Advance Care Directive	N	57 (97)	2 (3)
Increasing home safety to prevent accidents (e.g. falls, fires)	N	56 (95)	3 (5)
The potential benefits of being involved in research	N	46 (78)	13 (22)
Probable life expectancy, no matter how uncertain the information	N	37 (63)	22 (37)
At the time of diagnosis, people with dementia and their support person/s should be offered:			
Referral to relevant community organisations (e.g. Dementia Australia)	Y	59 (100)	0 (0)
Information about dementia in multiple formats, including written and web-based, according to their preferences	Y	58 (98)	1 (2)
A second consultation within 2 weeks of the initial diagnosis consultation to answer questions or discuss concerns.	N	31 (53)	28 (48)

^a^Items based on recommendations from the NHMRC Clinical Practice Guidelines and Principles of Care for People with Dementia [10]

^b^Percentages may not sum to 100% due to rounding

### Statistical analysis

Analysis was completed in STATA 11 [13]. Proportions of participants selecting each response were calculated for each item. For ease of interpretation, proportions of participants who responded “Strongly Agree” /“Agree” for each item were combined, as were proportions who responded “Strongly Disagree”/“Disagree”/“Unsure”.

## Results

Fifty-nine out of 140 eligible geriatricians completed the survey (42% response rate). The majority (69%, *n*=41) were consultant geriatricians. Participants had an average of 11.2 years’ experience (SD=9.2) and had seen an average of 26 people with dementia (SD=18.2) in the past month.

### Improving the accuracy and timeliness of dementia diagnosis

All participants agreed that increasing accessibility of geriatricians would improve accuracy and timeliness of diagnosis (see Table 1). Fewer (80%) agreed that making geriatrician consultations more affordable would improve these outcomes. Almost all agreed that training GPs in dementia diagnosis (97%) or incorporating standardised assessment for cognitive impairment in 75+ health assessments (92%) would improve accuracy and timeliness of diagnosis. Slightly fewer participants (85%) agreed that training practice nurses would be an effective strategy to improve these outcomes. Almost all (95%) agreed that timely and accurate diagnosis might be improved by increasing community awareness about the symptoms of dementia and what to do if symptoms are noticed.

### Provision of information at diagnosis

Most participants (97-100%) agreed that a range of information should be provided following diagnosis, including symptoms to expect in future, available treatment options, psychological support, and appointing substitute decision makers. Just over one third of participants (36%) did not think that information about life expectancy should be provided. All participants thought that referral to services supporting people with dementia in the community (e.g. Dementia Australia) should be offered. While the majority (98%) thought that that information should be provided in different formats according to patient preferences, just over half (53%) agreed that a second consultation should be offered within 2 weeks of the initial diagnosis to answer patient and support persons questions and discuss their concerns (see Table 1).

### Alignment with existing Australian clinical practice guidelines

Agreement with items that were based on the Australian clinical practice guidelines for the care of people with dementia [10] ranged from 88%-100% (see Table 1). A number of other strategies that are not currently included in Australian guidelines also received unanimous or majority support. For example, improving access to geriatricians and training general practitioners in accurately diagnosing dementia were recognised by 100 and 97% of participants respectively as potentially effective strategies for improving timely and accurate diagnosis. Between 95-100% of participants also supported provision of a range of information at diagnosis that is not currently included in guidelines, including lifestyle modifications that may improve health, appointing substitute decision makers, the benefits of making an Advance Care Directive and increasing home safety.

## Discussion

### Improving accuracy and timeliness of dementia diagnosis

Participants showed high levels of agreement with a range of possible patient, provider and system-level strategies for improving timely and accurate diagnosis of dementia. All agreed that strategies to improve accessibility of specialist care would likely improve rates of timely diagnosis. Lack of accessibility of specialist dementia care providers is a commonly cited barrier to providing timely and accurate diagnosis in Australia, particularly in rural areas [14, 15]. Although a large proportion of the population live in regional and rural areas, availability of geriatricians in these areas is limited, resulting in long wait times or the need to travel long distances [9]. Eighty percent of participants agreed that lowering the cost of specialist appointments may allow more opportunities for timely and accurate diagnosis. In Australia, although some of the consultation costs may be covered by government rebates and private health insurance coverage, there are often significant out-of-pocket expenses. These costs may be prohibitive for individuals of low socioeconomic background, leading to inequities in provision of timely diagnosis. In comparison, in some countries in Europe such as the Netherlands, there is universal insurance that allows for at least one memory clinic visit.

Participants also supported strategies to improve capacity for timely dementia diagnosis in primary care settings. Almost all supported the idea of additional training for general practitioners in accurately diagnosing dementia. A recent systematic review identified lack of training as one of the key barriers to improving general practitioners’ detection and diagnosis of dementia across a number of studies from the US and Europe [8]. Participants also agreed with the inclusion of a standardised measure of cognitive impairment as part of 75+ comprehensive health assessments. The 75+ health assessment is a comprehensive annual health check of older adults aged at least 75 years covering physical, emotional and social functioning. It is usually completed by a general practitioner or a combination of the general practitioner and practice nurse, and is covered by Australia’s universal health care insurance scheme [16]. While it is recommended that testing for possible cognitive impairment be included in the assessment, no standardised measure is recommended. Provision of a nationally-endorsed standardised measure may increase the likelihood of possible cases of dementia being detected as part of this assessment. Coupled with additional training in conducting thorough investigation of dementia symptoms, this may increase the capacity of primary care providers to identify and appropriately diagnose dementia.

At the patient level, a large majority of participants agreed that timely and accurate diagnosis would be improved by raising community awareness of dementia symptoms. A number of studies from the US and European countries including France, Germany and Ireland have found that seeking help for a possible diagnosis may be delayed due to a lack of knowledge about early symptoms and whether these were attributable to dementia [17]. Our recent study of 189 community members recruited from a hospital outpatient clinic in Australia (in preparation) showed variable levels of knowledge about dementia symptoms. While 87-96% were able to correctly identify memory-related symptoms (e.g. trouble remembering recent events), fewer (37-65%) correctly identified behavioural symptoms of dementia (e.g. aggression). By improving community awareness of the full range symptoms that might be experienced by individuals with dementia, this may reduce delays in individuals seeking assessment for possible dementia.

### Provision of information following dementia diagnosis

Participants agreed that information provided following a diagnosis of dementia should encompass a range of topics, including disease progression, current available treatment options and potential clinical trials, physical and psychological health, and advance care planning. However, observational studies have shown that the degree to which they involve patients in information provision about the diagnosis and implications of this is limited [18]. Accordingly, people with dementia and their support persons report suboptimal information provision following dementia diagnosis [12, 18], particularly regarding available local resources and potential legal implications (e.g. appointing a substitute decision maker). Support persons also have variable understanding of dementia and the symptoms which are attributable to this condition, impacting on their likelihood of seeking appropriate help for symptom management [12]. In our recent study of 169 Australian carers of people with dementia [in preparation], 37% reported they needed help with understanding which symptoms were caused by dementia. These findings suggest that additional strategies are needed to ensure that the individual diagnosed and their next of kin receive all relevant information and support.

Interestingly, there was near-unanimous agreement (97%) that people with dementia should be informed about the benefits and process of making an Advance Care Directive at diagnosis. Given the progressive nature of the disease, early engagement of people with dementia in this process may increase the likelihood that future medical care reflects the individual's wishes. However there has been limited exploration of preferences of people with dementia regarding timing of conversations on this topic [19]. There is also the question of who should initiate conversations about advance care planning. As the geriatrician is unlikely to have an ongoing role in the care of the person with dementia, it may be more appropriate for discussions about this topic to be initiated by other care providers with an established relationship with the patient, such as their primary care physician. Further investigation of the preferences of people with dementia regarding the timing of these conversations and who they believe would be most appropriate to introduce this issue are warranted.

Just under two-thirds of geriatricians were supportive of providing information about life expectancy at the time of diagnosis. Median life expectancy following dementia onset has been reported as varying between 3.3 to 11.7 years [20], and varies depending on a number of factors, such as age, gender, type of dementia, and degree of progression at diagnosis [21]. However, many support persons of people with dementia are unaware that the condition has any impact on length of life [22]. Advising people with dementia and their families about possible impacts on life expectancy may assist them in planning for the future. A systematic review showed that while many terminally ill patients would like to discuss life expectancy soon after diagnosis, some find discussion of this topic too soon after diagnosis distressing [23]. This suggests that people with dementia and their support persons should be consulted about whether they would prefer to have these conversations soon after diagnosis or at a later point.

Receiving a dementia diagnosis is highly distressing for both the individual diagnosed and their family, which may impact on understanding and retention of information provided [24]. Providing too much information at this time may contribute to ‘information overload’, which can increase the likelihood of confusion and misinterpretation of information [25], impair communication between patients and health care providers [26], and reduce patients’ intention to engage with educational materials [27]. However, only just over half of participants agreed that it would be beneficial to offer a second consultation following diagnosis. Cancer patients may prefer two shorter consultations to one longer consultation when making decisions about treatment [28]. Families of people with dementia perceive that follow-up sessions may be valuable as an opportunity to clarify understanding and reflect on the implications of a diagnosis [29]. Given the large amount of information that should be provided to the individual and their families following a diagnosis, offering a second consultation may also provide the clinician with an opportunity to reinforce previously provided information, provide additional information, and clarify any misunderstandings. The preferences of people with dementia and their families regarding this suggestion should be further explored.

### Alignment with clinical practice guidelines

Reassuringly, there was a high degree of support from geriatricians for strategies that were derived from the Australian clinical practice guidelines for the care of people with dementia [10]. However, there was also support for a range of other strategies put forward by the Expert Advisory Group that are not currently included in guidelines. For example, strategies to improve general practitioners’ capacity to provide timely and accurate diagnosis were supported by over 90% of participants. The guidelines recommend that following diagnosis, information is provided about symptoms of dementia, available treatments, and how to access social support. While participants agreed with these recommendations, there was also support for provision of a range of other information at this time, including lifestyle modifications to improve health, and information about advance care planning. While advance care planning is recommended in the guidelines, there is no clear direction about when these discussions should take place. The current findings suggest that geriatricians perceive it may be appropriate for these discussions to occur soon after diagnosis. These findings may be considered for incorporation in future iterations of the guidelines.

### Limitations and future directions

The response rate for this study was 42%, which is comparable with surveys of other health care provider groups [30–32] such as general practitioners. Those who consented to participate may be more interested in this topic and therefore more likely to agree with the suggested strategies. As there is no standard approach for diagnosing and following up on a diagnosis of dementia in Australia and the study was conducted in only one Australian state, this may limit the generalisability of findings to other states. In future the survey could be expanded to include other Australian states, and even other countries to explore generalisability. We did not include any open-ended questions asking geriatricians for additional suggestions for strategies to improve care; this may have yielded further ideas beyond the scope of those suggested by our Expert Advisory Group.

This study focuses on geriatricians as they play a central role in the diagnostic process. However geriatricians only see those patients that primary care providers decide to refer on. These patients may be likely to have earlier stage symptoms requiring further specialist assessment to determine whether a diagnosis is warranted. A comparison of geriatricians’ views with the perspectives of other providers involved in providing a diagnosis and follow up care (e.g. primary care physicians and nurses) would be extremely valuable. Following this, studies are needed to assess the degree to which people with dementia and their families receive care in alignment with the recommended strategies.

## Conclusions

Geriatricians were supportive of a range of suggested strategies for improving quality of care at diagnosis. These strategies should be considered for highlighting and/or inclusion in future versions of national dementia care guidelines and training packages for providers involved in the diagnosis of people with dementia.

## Supplementary Information

**Additional file 1.** Copy of the participant survey.

## Data Availability

The datasets generated and/or analysed during the current study are not publicly available as this is not allowable under our ethics approval, but are available from the corresponding author on reasonable request.

## Abbreviation

ANZSGM: Australian and New Zealand Society for Geriatric Medicine
