# Supplementary material for: Optimising diagnosis and post-diagnostic support for people living with dementia: geriatricians’ views
Source: BMC Geriatr. 2022 Feb 19;22:143. doi: 10.1186/s12877-022-02814-0 (PMC8858511; doi:10.1186/s12877-022-02814-0)
Supplement: Supplementary file 1 — Additional file 1. Copy of the participant survey. [file 12877_2022_2814_MOESM1_ESM.docx]

**Optimal care for dementia diagnosis**

We are seeking your expert opinion about what should constitute optimal care for diagnosis of dementia.  **Please indicate the extent to which you agree with the following statements.**

| To improve the accuracy and timeliness of dementia diagnosis there is a need for: | Strongly Agree | Agree | Disagree | Strongly Disagree | Unsure |
| --- | --- | --- | --- | --- | --- |
| 1. General practitioners to receive training in accurately diagnosing dementia | 1 | 2 | 3 | 4 | 5 |
| 1. Involvement of dementia-trained general practice nurses in the diagnosis of dementia | 1 | 2 | 3 | 4 | 5 |
| 1. 75+ health assessments for general practice patients to include a standardised measure of cognitive impairment | 1 | 2 | 3 | 4 | 5 |
| 1. Undergraduate medical students to receive training and rigorous skills assessment for dementia diagnosis | 1 | 2 | 3 | 4 | 5 |
| 1. Increased community awareness about early symptoms of dementia and what to do if symptoms are noticed | 1 | 2 | 3 | 4 | 5 |
| 1. Individuals to have access to a geriatrician in their local area | 1 | 2 | 3 | 4 | 5 |
| 1. Consultations with geriatricians to be more affordable | 1 | 2 | 3 | 4 | 5 |

| At the time of diagnosis, people with dementia and their support person/s should be provided with information about: | Strongly Agree | Agree | Disagree | Strongly Disagree | Unsure |
| --- | --- | --- | --- | --- | --- |
| 1. Symptoms of dementia which may occur in future | 1 | 2 | 3 | 4 | 5 |
| 1. Probable life expectancy, no matter how uncertain the information | 1 | 2 | 3 | 4 | 5 |
| 1. The benefits and risks of available treatment options | 1 | 2 | 3 | 4 | 5 |
| 1. Lifestyle modifications that may improve health or quality of life | 1 | 2 | 3 | 4 | 5 |
| 1. How to access psychological support or counselling | 1 | 2 | 3 | 4 | 5 |
| 1. Appointing a substitute decision maker(s) (e.g. Enduring Guardian and Enduring Power of Attorney) | 1 | 2 | 3 | 4 | 5 |
| 1. The benefits and process of making an Advance Care Directive | 1 | 2 | 3 | 4 | 5 |
| 1. Increasing home safety to prevent accidents (e.g. falls, fires) | 1 | 2 | 3 | 4 | 5 |
| 1. The potential benefits of being involved in research | 1 | 2 | 3 | 4 | 5 |

| At the time of diagnosis, people with dementia and their support person/s should be offered: | Strongly Agree | Agree | Disagree | Strongly Disagree | Unsure |
| --- | --- | --- | --- | --- | --- |
| 1. Information about dementia in multiple formats, including written and web-based, according to their preferences | 1 | 2 | 3 | 4 | 5 |
| 1. Referral to relevant community organisations (e.g. Dementia Australia) | 1 | 2 | 3 | 4 | 5 |
| 1. A second consultation within 2 weeks of the initial diagnosis consultation to answer questions or discuss concerns. | 1 | 2 | 3 | 4 | 5 |

**Demographics**

| **1.** | Are you a: | 1. Consultant geriatrician 2. Advanced trainee 3. Other (please specify)   _____________________________ |
| --- | --- | --- |
| **2.** | How long have you been practising as a geriatrician? | _________________________years |
| **3.** | In the past month, approximately how many people with dementia have you seen professionally? | _________________________ |

**You have now completed the survey. Thank you very much for your time.**

Please place your completed survey into the envelope provided and return to

Rob Sanson-Fisher during the conference

Email: Rob.Sanson-Fisher@newcastle.edu.au
